# 84 Adapting the Recurrent Urinary Tract Infection Impact Questionnaire (RUTIIQ) for Populations with Neurogenic Bladder through Cognitive

**DOI:** 10.1017/ash.2026.10510

**Published:** 2026-06-23

**Authors:** Lisa Hall, Sara Reese, Elizabeth Monsees, Annie Wirtz, Monika Pogorzelska-Maziarz

**Affiliations:** 1 The University of Queensland; 2 APIC; 3 Children’s Mercy Hospital; 4 Children’s Mercy Kansas City

## Abstract

**Background:** Over the past decade there has been an increased focus on infection prevention in post-acute care settings, including long-term care (LTC), skilled nursing facilities (SNF), rehabilitation facilities and home health. Many of these settings provide care for older people with complex care needs. Research has shown high rates of inappropriate antibiotic prescribing, with sub-optimal drug choice, dosing and duration – all targets for antimicrobial stewardship programs (ASP). Although national guidance is available, implementation remains challenging. In this study, we explored the structure and leadership of ASP in a variety of post-acute care settings from an infection prevention perspective. **Methods:** Infection Preventionists (IPs) were invited to participate in the 5-yearly Association for Professionals in Infection Prevention (APIC) Megasurvey. The electronic survey was advertised using snowball recruitment by APIC between June 6 and July 31, 2025. Data were collected on ASP characteristics, including structure and leadership of programs, and resources available to support appropriate prescribing. Descriptive statistics were used to summarize the data. **Results:** A total of 489 respondents reported working in post-acute settings. Of these 239 (49%) worked in long term care, 200 (41%) in skilled nursing facilities, 26 (5%) in inpatient rehabilitation and 24 (5%) in home health. Facility-based post-acute care IPs overwhelmingly reported the existence of ASPs (LTC-82%; SNF/Rehabilitation – 93%) with a committee having oversight (LTC-86%; SNF/Rehabilitation – 80%). This governance function was often incorporated into infection prevention committees (LTC - 52%; SNF/Rehabilitation – 43%). In LTC, ASPs were led by a range of healthcare professionals including IPs (27%), infectious diseases (ID) physicians (10%), ID pharmacists (10%), pharmacists (non-ID) (23%), and other medical professionals such as internists (22%). In SNFs/ Rehabilitation facilities, a similar pattern was observed. IPs reported they had up-to-date recommendations available for infection management (LTC - 88%, SNF/Rehabilitation -83%), and around half also noted existence of regular rounds to allow inquiry about appropriate antibiotic prescribing (LTC - 52%, SNF/Rehabilitation - 53%) Only 29% of IP respondents from home health reported having an ASP. Although numbers are small, it appears that 70% of these programs had the governance of an ASP Committee but there was little IP involvement in leadership or antibiotic prescribing review activities. **Conclusion:** This study provides unique insights into ASP in post-acute care. Programs are now well-established in many facility types. More work is needed to understand and help IPs develop ASP expertise relevant for post-acute settings, particularly in home health.

